# Pharmacological treatment of hypoactive delirium in critically ill patients: a systematic review

**DOI:** 10.1186/cc12652

**Published:** 2013-06-19

**Authors:** JPLM Carvalho, R Alvim, J Martins, C Bouza, P Zenaide, R Zantieff, B Pondé, D Amorim, LC Quarantini, D Gusmao-Flores

**Affiliations:** 1 Faculdade de Medicina da Bahia, Universidade Federal da Bahia, Salvador, BA, Brazil; 2 Escola Bahiana de Medicina e Saúde Pública, Salvador, BA, Brazil; 3 University Hospital Prof. Edgard Santos, Universidade Federal da Bahia, Salvador, BA, Brazil

## Introduction

Delirium is generally managed by treating its underlying causes. However, symptomatic treatment may also be indicated. Although hypoactive delirium in critically ill patients is the most prevalent subtype of delirium, the effects of treatment with drugs specifically for this group are not well defined. The aim of this systematic review is to evaluate the role of pharmacological treatment in critically ill patients with hypoactive delirium.

## Methods

A systematic review was conducted, based on the PRISMA criteria, to identify articles on the pharmacological approach to hypoactive delirium in critically ill patients. First, a MEDLINE and SciELO database search was performed for articles published in the English language, involving patients in ICUs in which pharmacological therapy was used to treat delirium. Second, these studies were reevaluated to identify subtypes of delirium and the impact of the treatment.

## Results

The number of studies included in the qualitative synthesis was 18. One-half of them were clinical trials and the others were either letters or comments. However, only one study specified the treatment of hypoactive subtype delirium. The design of this study was a *post-hoc *analysis of a double-blind, randomized, placebo-controlled study that used quetiapine as an adjuvant therapy to haloperidol. This study suggested that quetiapine appears to have more rapid resolution of many delirium symptoms, including a hypoactive state. These results were not statistically significant. The other 17 studies do not address the subtype.

## Conclusion

There is poor evidence regarding the use of drugs for the management of hypoactive delirium. Not only the study design but the number of patients studied in the single trial is very limited, which affects the power of evidence. Double-blind, randomized, placebo controlled trials must be performed to guide the treatment and the management of hypoactive delirium.
